# Additional role of second washing specimen obtained during single bronchoscopy session in diagnosis of pulmonary tuberculosis

**DOI:** 10.1186/1471-2334-13-404

**Published:** 2013-09-02

**Authors:** Hongseok Yoo, Jae-Uk Song, Won-Jung Koh, Kyeongman Jeon, Sang-Won Um, Gee Young Suh, Man Pyo Chung, Hojoong Kim, O Jung Kwon, Nam Yong Lee, Sookyoung Woo, Hye Yun Park

**Affiliations:** 1 Division of Pulmonary and Critical Care Medicine, Department of Medicine, Samsung Medical Center, Sungkyunkwan University School of Medicine, 81 Irwon-ro, Gangnam-gu, Seoul, Republic of Korea; 2 Division of Pulmonary and Critical Care Medicine, Department of Medicine, Kangbuk Samsung Hospital, Sungkyunkwan University School of Medicine, Seoul, Republic of Korea; 3 Department of Laboratory Medicine and Genetics, Samsung Medical Center, Sungkyunkwan University School of Medicine, Seoul, Republic of Korea; 4 Biostatistics Team, Samsung Biomedical Research Institute, Seoul, Republic of Korea

**Keywords:** Bronchial washing, Bronchoscopy, Diagnosis, Tuberculosis

## Abstract

**Background:**

Flexible bronchoscopy with bronchial washing is a useful procedure for diagnosis of pulmonary tuberculosis (TB), when a patient cannot produce sputum spontaneously or when sputum smears are negative. However, the benefit of gaining serial bronchial washing specimens for diagnosis of TB has not yet been studied. Therefore, we conducted a retrospective study to determine the diagnostic utility of additional bronchial washing specimens for the diagnosis of pulmonary TB in suspected patients.

**Methods:**

A retrospective analysis was performed on 174 patients [sputum smear-negative, n = 95 (55%); lack of sputum specimen, n = 79 (45%)] who received flexible bronchoscopy with two bronchial washing specimens with microbiological confirmation of pulmonary TB in Samsung Medical Center, between January, 2010 and December, 2011.

**Results:**

Pulmonary TB was diagnosed by first bronchial washing specimen in 141 patients (81%) out of 174 enrolled patients, and an additional bronchial washing specimen established diagnosis exclusively in 22 (13%) patients. Smear for acid-fast bacilli (AFB) was positive in 46 patients (26%) for the first bronchial washing specimen. Thirteen patients (7%) were positive only on smear of an additional bronchial washing specimen. Combined smear positivity of the first and second bronchial washing specimens was significantly higher compared to first bronchial washing specimen alone [Total cases: 59 (34%) vs. 46 (26%), p < 0.001; cases for smear negative sputum: 25 (26%) vs. 18 (19%), p = 0.016; cases for poor expectoration: 34 (43%) vs. 28 (35%), p = 0.031]. The diagnostic yield determined by culture was also significantly higher in combination of the first and second bronchial washing specimens compared to the first bronchial washing. [Total cases: 163 (94%) vs. 141 (81%), p < 0.001; cases for smear negative sputum: 86 (91%) vs. 73 (77%), p < 0.001; cases for poor expectoration: 77 (98%) vs. 68 (86%), p = 0.004].

**Conclusions:**

Obtaining an additional bronchial washing specimen could be a beneficial and considerable option for diagnosis of TB in patients with smear-negative sputum or who cannot produce sputum samples.

## Background

Pulmonary tuberculosis (TB) is a major public health challenge worldwide, with an estimated 8.8 million new cases and 1.45 million TB-related deaths in 2010 [1]. Although various diagnostic tests for pulmonary TB have been developed, its definitive diagnosis still relies on culturing *Mycobacterium tuberculosis* (*M*. *tuberculosis*) from respiratory secretions. For sputum samples, international guidelines recommend obtaining at least two specimens from patients with suspected tuberculosis to increase the diagnostic sensitivity for identification of *M*. *tuberculosis*[2]. In patients with negative sputum smear or who cannot produce sputum samples, previous studies have proved efficacy of an alternative method of obtaining respiratory samples by bronchoscopy in diagnosis of TB [3-8]. However, it is yet known whether an additional bronchial specimen acquired from bronchial washing has an incremental diagnostic yield for diagnosis of TB as second and third sputum samples have [9-11]. Therefore, the aim of this study was to assess the diagnostic value of the second bronchial washing sequentially obtained after the first bronchial washing specimen in suspected pulmonary TB patients who are unable to expectorate or have pre-bronchoscopy smear-negative sputum.

## Methods

### Study population

This was a retrospective observational study on the role of additional bronchial washing specimens in diagnosis of TB in Samsung Medical Center (a 1960-bed, university-affiliated, tertiary referral hospital in Seoul, South Korea) between January 2010 and December 2011. TB case is clinically suspected as a patient currently not receiving tuberculosis treatment with a persistent cough for >3 weeks or symptoms consistent with TB (chest pain, low-grade fever, night sweats, shortness of breath, and weight loss) with chest radiograph or computed tomograph scan suggestive of pulmonary TB. In clinically suspected TB patients with smear-negative sputum or who are difficult to produce sputum samples, we obtain three serial bronchial washing samples during bronchoscopy.

**Figure 1:**
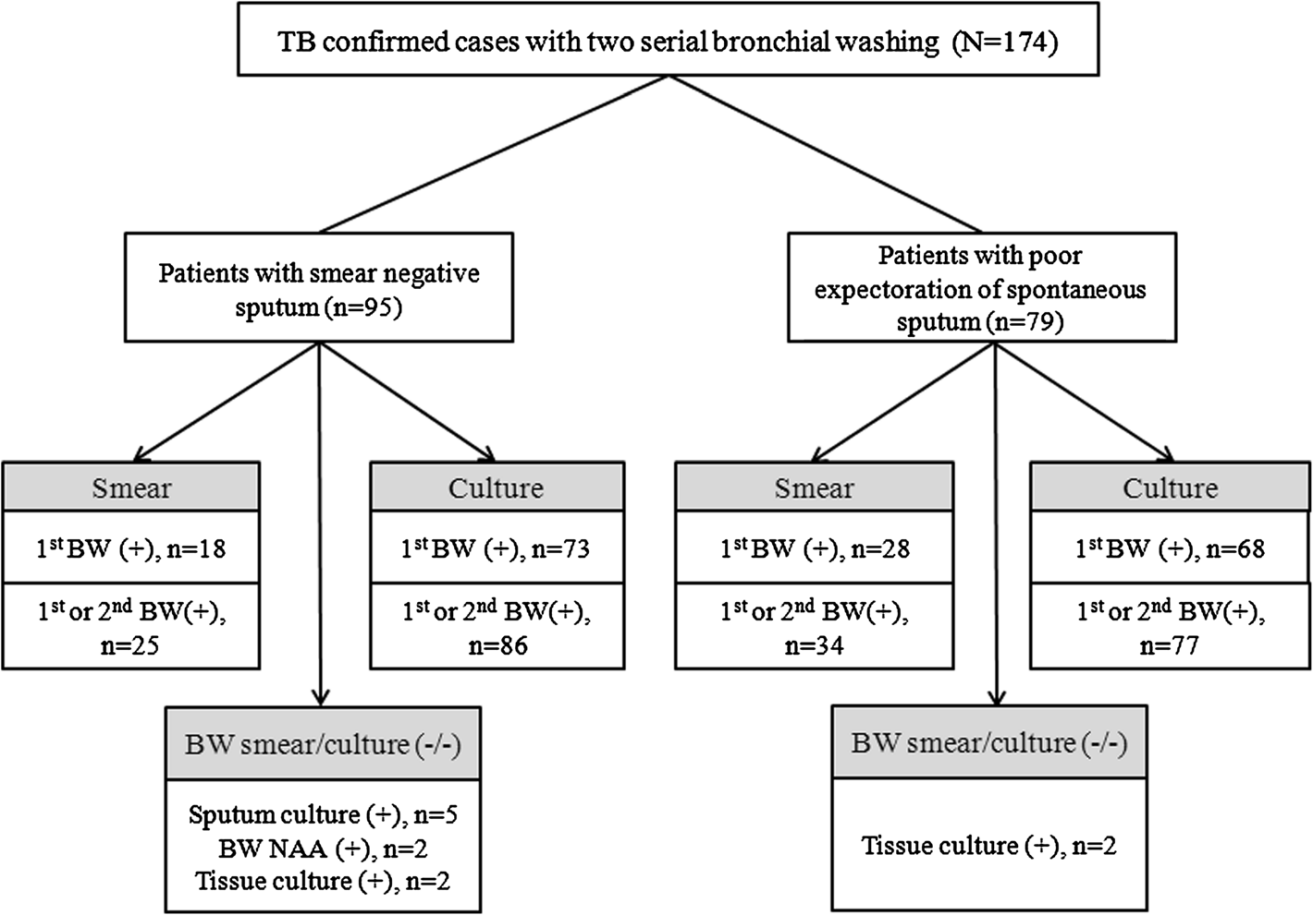
**Diagnostic flow of 174 study patients.** BW, bronchial washing; NAA = nucleic acid amplification.

Of those who underwent bronchoscopy with two bronchial washing specimens for AFB smear and culture, we recruited 225 patients with a microbiological confirmation of *M*. *tuberculosis* during the study period. Of those patients, 51 receiving tuberculosis treatment before bronchoscopy were excluded from the study. This left a population of 174 patients with a diagnosis of pulmonary tuberculosis from bronchial washing (n = 163); other methods, such as pathologic confirmation with culture from tissue obtained by bronchoscopy, (n = 4); nucleic acid amplification assay for *M*. *tuberculosis* on the third bronchial washing (n = 2); and sputum culture (n = 5) (see Figure 1). Demographic, clinical and radiological variables were determined by retrospective analysis of medical records. The Institutional Review Board of Samsung Medical Center approved the review and publication of data obtained from the patients’ records (Protocol No: 2011-07-037) and the study was conducted in compliance with the Helsinki Declaration. Informed consent was waived due to the retrospective nature of this study.

### Bronchoscopy procedures

Bronchoscopy with bronchial washing was performed according to the standard technique as described previously [12,13]. Patients were sedated with intravenous midazolam. Under conscious sedation, the bronchoscope (Olympus, Tokyo, Japan) was inserted trans-nasally or -orally and lidocaine of 5 mL to 10 mL as needed was sprayed intratracheally. After placing bronchoscope at the targeted segment, bronchial washing was performed by instilling 10 mL of warm sterile saline. Several breaths were allowed for the fluid to pool at the target segment before retrieval. This procedure was repeated twice at the same segment. Each retrieved sample was labeled as the first, second, and third sample. First and second samples were then tested for acid-fast bacilli (AFB) by smear and culture. The third specimen was subjected to a nucleic acid amplification assay for *M*. *tuberculosis*[14,15]. When multiple pulmonary infiltrates were visible on chest radiography, bronchial washing was performed at the segment exhibiting the most severe abnormalities.

### Microbiological examination

Aliquots of specimens were decontaminated with the 2% N-acetyl-L-cysteinhydroxide (NALC-NaOH). Then the specimens were stained by auramine-rhodamine fluorescent and Ziehl-Neelsen stain and examined under microscopy. The processed specimens were also plated onto both 3% Ogawa solid medium (Shinyang, Seoul, Korea) and liquid culture medium (MGIT 960 system, Becton Dickinson, Sparks, MD, USA) and were incubated at 36°C for 6 weeks. Nucleic acid amplification test for *M*. *tuberculosis* was performed using COBAS TaqMan MTB assay (Roche Diagnostics, Basel, Switzerland) according to the manufacturer’s guideline.

### Definition

Microbiological confirmation of *M*. *tuberculosis* was defined as growth of *M*. *tuberculosis* or positive test result of the nucleic acid amplification for *M*. *tuberculosis* from respiratory or tissue samples. Immunocompromised condition was defined as following: undergoing chemotherapy for an malignancy within 4 weeks, being seropositive for human immunodeficiency virus or undergoing daily administration of systemic corticosteroids (at least 20 mg prednisolone or equivalent for more than 1 month), combination therapy with low dose corticosteroids and other immunosuppressants including azathioprine, mycophenolate, methotrexate, cyclosporine, or cyclophosphamide or under status of solid or hematologic transplantation.

### Statistical analysis

All data are presented as numbers (percentages) for categorical variables and as medians with interquartile ranges (IQR) for continuous variables. The diagnostic yield of repeated bronchial washing for the diagnosis of TB was determined. The diagnostic consistency according to the number of bronchial washing was analyzed using the McNemar’s test. Diagnostic yield and diagnostic consistency were also sub-analyzed based on the reason of bronchoscopy. To identify the factors to predict diagnostic yield according to the number or order of bronchial washing specimens, clinical and laboratory characteristics according to positivity of first and/or second bronchial washing specimens were compared using the Kruskal-Wallis test for continuous variables and the χ2 test for categorical variables. Statistical analyses were performed using IBM SPSS Statistics 21.0 (IBM, Chicago, IL, USA), and two-sided *P*  <  0.05 was considered significant.

The incremental yield of an additional bronchial washing specimen was defined as [(number of cases additionally diagnosed by second bronchial washing specimen/number of all diagnosed cases) × 100] [16].

## Results

The baseline characteristics of the patients are summarized in Table 1. There were 90 males and 84 females, with a median age of 53 (IQR 34–68) years. Seventy six (44%) patients had one or more comorbidities and 32 (18%) had undergone previous TB treatment. The most common presenting symptoms were cough (n = 43, 25%) and sputum (n = 23, 13%). Chest radiographs were available for analysis in all patients. The most common radiologic finding was small nodules (n = 95, 55%), followed by consolidation (n = 45, 26%), atelectasis (n = 19, 11%), and cavitation (n = 18, 10%). The most common sites at which bronchial washing was performed were the right upper lobe (n = 71, 41%) and the left upper lobe (n = 49, 28%). Regarding adverse events, five patients (2.9%) had fever after bronchoscopy and one (0.6%) had tachycardia, which resolved after bronchoscopy.

**Table 1:** Baseline characteristics of 174 study patients

	**No. of patients (%) or median (IQR)**
Age, years	53 (34 – 68)
Gender, female	84 (48)
Comorbidity	76 (44)
Malignancy	31 (18)
Cardiovascular disease	29 (17)
DM	25 (14)
Chronic hepatitis	3 (2)
Respiratory disease	4 (2)
Rheumatic disease	3 (2)
Transplantation	2 (1)
Others*	6 (3)
Smoking	
Ex-smoker	26 (15)
Current smoker	41 (24)
Previous history of TB treatment	32 (18)
Symptoms	75 (43)
Cough	43 (25)
Sputum	23 (13)
Fever	15 (9)
Hemoptysis	11 (6)
Chest pain	9 (5)
Dyspnea	7 (4)
Sweating	2 (1)
Chest radiology	
Small nodules	95 (55)
Consolidation	45 (26)
Atelectasis	19 (11)
Cavitation	18 (10)
Mass	14 (8)
Effusion	9(5)
Fibrotic scar	8 (5)
Normal	3 (2)
Location of bronchial washing	
Right upper lobe	71 (41)
Right middle lobe	19 (11)
Right lower lobe	25 (14)
Left upper lobe	49 (28)
Left lower lobe	10 (6)

Abbreviations: *IQR*, interquartile ranges; *DM*, diabetes mellitus; *TB*, tuberculosis.

* Hyperthyroidism (n = 2), hypothyroidism (n = 1), paraplegia due to spinal cord injury (n = 1), Klippel-Feli syndrome (n = 1), osteoporosis (n = 1).

Categorical variables were denoted by No. of patients (%).

Continuous variable was denoted by median (IQR).

**Figure 2:**
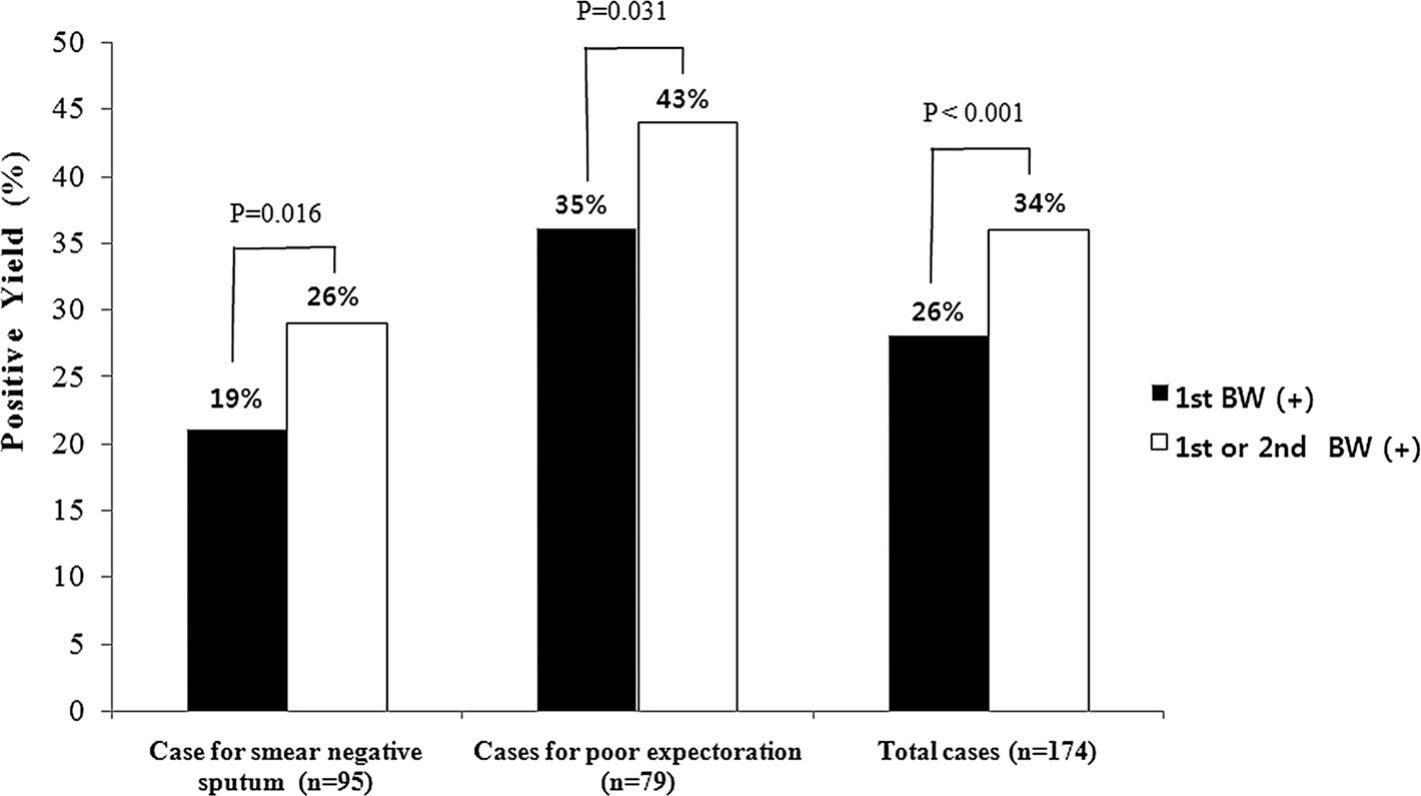
**Smear positive yield of single bronchial washing and two consecutive bronchial washing specimens.** BW, bronchial washing.

**Figure 3:**
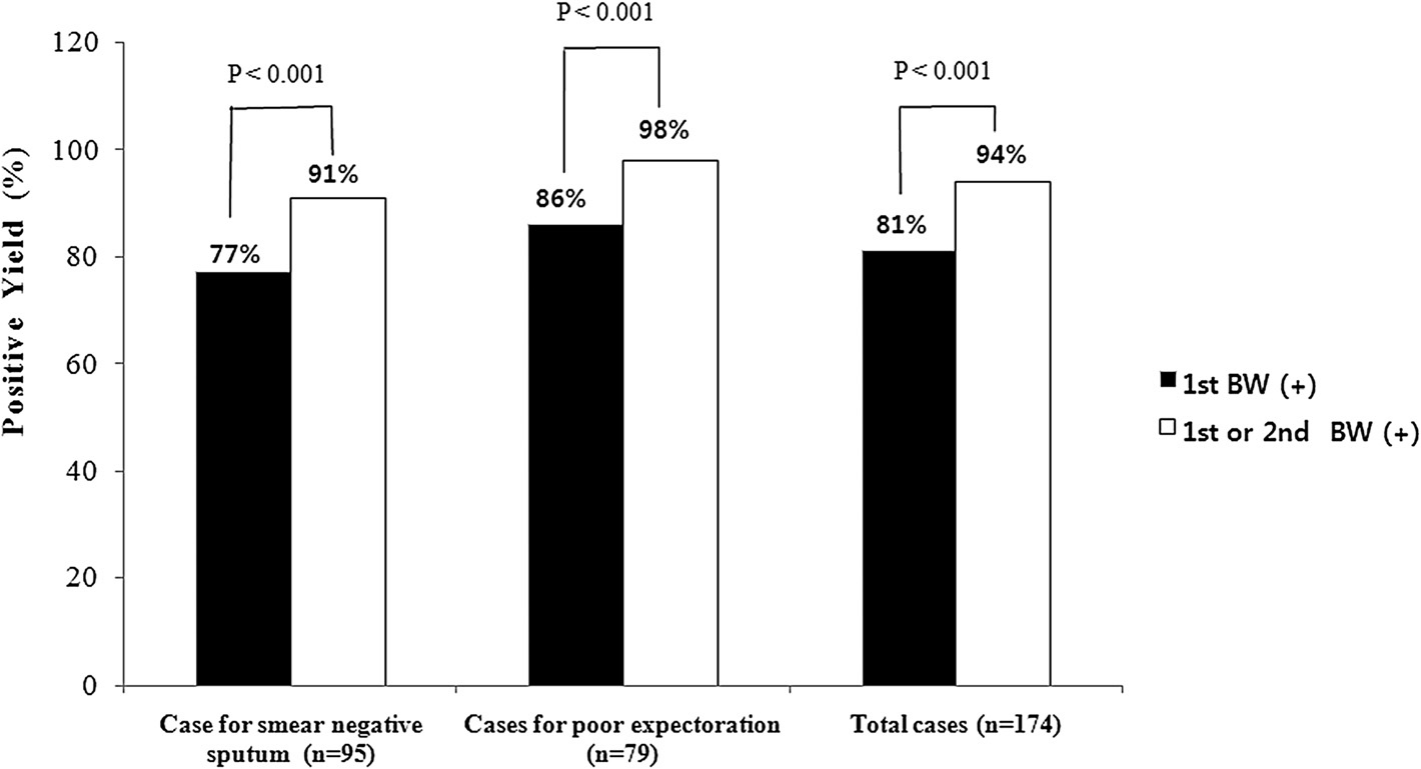
**Culture positive yield of single bronchial washing and two consecutive bronchial washing specimens.** BW, bronchial washing.

Out of 174 study patients, 46 (26%) patients had a positive AFB smear in the first bronchial washing specimen, and additional 13 (7%) patients had a positive AFB smear only in the second bronchial washing specimen. Nucleic acid amplification assay for *M*. *tuberculosis* using the third bronchial washing was positive in 72 (41%) patients and was the sole method to diagnose pulmonary TB in 2 patients. Combined smear positivity of the first and second bronchial washing specimens was significantly higher than that of the first bronchial washing specimen alone [59 (34%) vs. 46 (26%), p < 0.001]. (Figure 2) On culture, *M*. *tuberculosis* was identified in 141 (81%) patients by first bronchial washing specimen. Additional bronchial washing specimen identified *M*. *tuberculosis* exclusively in 22 (13%) patients. The diagnostic yield of culture was also significantly higher in combination of the first and second bronchial washing specimens compared to the first bronchial washing [163 (94%) vs. 141 (81%), p < 0.001]. (Figure 3).

Diagnostic yield was assessed based on the reason of bronchoscopy. Out of 95 patients with smear-negative sputum, 18 patients (19%) had a positive AFB smear in the first bronchial washing and an additional seven (7%) had a positive AFB smear from the second bronchial washing. Combined smear positivity of the first and second bronchial washing specimens was higher than that of first bronchial washing specimen alone [25 (26%) vs. 18 (19%), p = 0.016]. (Figure 2) On culture, *M*. *tuberculosis* was identified in 73 patients from the first bronchial washing and 86 patients had pulmonary TB on culture from combination of the first and second bronchial washing [86 (91%) *vs*. 73 (77%), *p* < 0.001]. (Figure 3) The incremental yield of culture positive cases was 14% with the additional second bronchial washing. Of 79 patients who were unable to expectorate, an AFB smear by direct microscopy was positive in 28 patients (35%) for the first bronchial washing and six (8%) were positive upon an additional bronchial washing smear. Cultures positive for *M*. *tuberculosis* were obtained in 68 patients (86%) using first bronchial washing specimens, and the first or second bronchial washing specimen was diagnostic in 77 patients (98%). Accordingly, the incremental yield from the second bronchial washing was 11%. The diagnostic yield for smear and culture of the combined bronchial washing specimens were significantly higher compared to the first bronchial washing specimen [smear: 34 (43%) vs. 28 (35%), p = 0.031; culture: 77 (98%) vs. 68 (86%), p < 0.001]. (Figure 2 and 3).

A drug susceptibility test was performed in 172 patients, excluding two who were diagnosed by a positive nucleic acid amplification test without culture of *M*. *tuberculosis*. Of 141 patients first bronchial washing of whom was cultured, 20 had drug-resistant tuberculosis (13 had multi-drug resistance (MDR) and seven had isoniazid (INH) resistance (INH-R)). The second bronchial washing identified seven additional patients with drug–resistant tuberculosis (four with MDR including one with extensive drug-resistance (XDR) and three with INH–R).

Table 2 shows comparisons of the clinical and radiological characteristics of patient groups with a positive result only on the first bronchial washing, only on the second bronchial washing, and in both bronchial washings (n = 163). There were no significant factors to predict diagnostic yield according to the number or order of bronchial washing specimens, including age, gender, comorbidities, smoking history, previous TB history or immune status. There were also no significant differences among groups regarding radiographic findings, as well.

**Table 2:** Comparisons of the clinical and radiological characteristics according to positive result in serial bronchial washing specimens in 163 patients*

	**BW1 (+) (n = 30)**	**BW2 (+) (n = 22)**	**BW1 &2 (+) (n = 111)**	**P value**
Age, years	54 (34 – 71)	60 (29 – 71)	52 (34 – 68)	0.898
Gender, female	11 (37)	12 (54)	57 (51)	0.310
Presence of Comorbidity	11 (37)	10 (46)	51 (46)	0.657
Immunocompromised patients	1 (3)	1 (5)	11 (10)	0.407
Current of ex- smoker	12 (40)	9 (41)	42 (38)	0.951
Previous history of treatment TB	5 (17)	4 (18)	23 (21)	0.869
Clinical Symptom	13 (43)	8 (36)	51 (46)	0.707
No symptoms	17 (57)	14 (64)	60 (54)	0.707
Chest radiology				
Fibrotic scar	1 (3)	0	6 (5)	0.500
Cavitation	3 (10)	5 (23)	10 (9)	0.169
Nodules	16 (53)	17 (77)	58 (52)	0.093
Mass	5 (17)	0	7 (6)	0.057
Consolidation	6 (20)	3 (14)	34 (31)	0.174
Atelectasis	1 (3)	3 (14)	13 (12)	0.358
Effusion	1 (3)	1 (5)	6 (5)	0.894
Drug resistance	3 (10)	7 (32)	17 (15)	0.092

*BW*, Bronchial washing.

Categorical variables were denoted by No. of patients (%).

Continuous variable was denoted by median (IQR).

*Patients with positive results for *M*. *tuberculosis* from bronchial washing.

## Disscusion

To our knowledge, this is the first study of the diagnostic value of an additional bronchial specimen acquired after the first bronchial washing. This retrospective study demonstrated that the diagnosis of pulmonary tuberculosis was increased by up to 13% with second bronchial washings in subjects whose spontaneous sputum was smear negative or who could not produce spontaneous sputum. In addition, second bronchial washing specimen identified additional drug-resistant *M*. *tuberculosis* in 7 patients (four MDR pathogens including one XDR and three INH-R pathogens).

It is well recognized that the smear positivity yield in sputum increases as the number of sputum samples increases in patients suspected as having pulmonary TB. A large study conducted in Tanzania showed that an incremental yield of 83.4% with the first, 12.2% with the second, and 4.4% with the third smear was estimated for the total number of expected cases (combined suspects with a complete set of three sputum smears examined and one with an incomplete examination) [11]. In Malawi, all TB suspects submit three sputum specimens for smear examination and the diagnosis is made by the first specimen in 83%, the second specimen in 13%, and the third specimen in 4% of patients [17]. Given the high diagnostic yield of over 10% in the second sputum smear [18] and the cost-effectiveness of obtaining the third specimen in low-income countries, [19] international guidelines recommend to obtain at least two sputum specimens from patients with suspected tuberculosis to increase the diagnostic yield for identification of *M*. *tuberculosis*[2]. In line with previous studies that used serial sputum samples, [11,17,18] our findings demonstrated for the first time a 13% culture incremental yield from an additional bronchial washing compared with performing only a single bronchial washing in patients with smear-negative sputum or in those in whom sputum was unavailable. Although no other reports or universal cut-off values are available for comparison with our results, we believe that an incremental culture rate of 13% for the second bronchial washing highlights the importance of additional bronchial washings.

In our study, consecutive first and second bronchial washings provided high TB diagnostic yield in patients who were negative on sputum smear microscopy or were unable to expectorate sputum; culture for *M*. *tuberculosis* was positive in 163 of 174 patients (94%). When our patients were divided according to the reason for performance of bronchoscopy, 77% of those who were negative on sputum smear microscopy were culture-positive in the first bronchial washing, which is consistent with previous reports of a positivity rate of 65-78% for culture of a single bronchial washing [7,8]. Use of a second bronchial washing added 8% to the positivity rate of culture for diagnosis of pulmonary TB, leading to a diagnostic yield of 85% of suspected TB patients with smear-negative sputum. In patients who were unable to expectorate sputum, however, few studies have focused on the diagnostic power of bronchial washing, while our data showed that consecutive first and second bronchial washings led to a rapid diagnosis in 43% by microscopy smear and accounted for the culture positivity of 77 of 79 patients (98%). Thus, these data indicate the diagnostic importance of bronchial washing in patients who lack spontaneous sputum for TB diagnosis.

In our study, the yield of *M*. *tuberculosis* culture was almost three times that of AFB smears, confirming the importance of culture for the diagnosis of pulmonary TB in our patients. In an era of increasing drug resistance, isolation of *M*. *tuberculosis* from respiratory specimens is important for drug sensitivity test. Additional 7 (26% of all drug- resistant TB) patients have been confirmed to have drug-resistant tuberculosis in the present study by means of further secondary bronchial washing. It indicates that an additional bronchial washing is a meaningful process not only in increasing the diagnostic yield but also in obtaining *M*. *tuberculosis* strain which allows drug susceptibility test.

For patients with positive results for *M*. *tuberculosis* from bronchial washing, there are no specific clinical or radiologic parameters which can determine who needs second bronchial washing. The reported adverse events associated with the procedure were tolerable. Furthermore, the inhibitory effect of lidocaine on the growth of *M*. *tuberculosis* might contribute to the false negative rate and/or relatively low colony counts of *M*. *tuberculosis* from first bronchial washing specimens [20]. Thus, we suggest that obtaining two serial bronchial samples is safe and reasonable during performance of bronchoscopy in patients with smear-negative sputum or in those who cannot produce sputum samples.

To fully appreciate these results, the limitations of this study must be acknowledged. First, given the retrospective design of this study, it is possible that selection bias influenced our findings. A prospective study would be needed to fully validate these promising findings. Additionally, the increased yield of TB diagnosis in our study might have been related to larger cumulative volume rather than the repeated washing process. A prospective study comparing separate bronchial washings and single bronchial washing with the same amount of total saline is required to address this question. Third, our study was conducted at a single institution in an area of intermediate TB burden (incidence of TB, 97 per 100,000), which might limit the application of our findings, especially in areas of differing TB prevalence. Finally, we were unable to evaluate the economic cost associated with the study protocol or the counterproductive effects of overburdened technicians. High costs and technician shortages may limit the use of bronchoscopy in other situations.

## Conclusions

In summary, we found that obtaining additional second bronchial washing sample increased the diagnostic yield by up to 13% under tolerable adverse events. This suggests that two serial bronchial washings are a beneficial and reasonable option for detection of TB and evaluation of drug resistance in patients who have negative AFB smears or who are unable to produce sputum samples in intermediate TB-burden countries.

## Abbreviations

AFB: Acid-fast bacilli; M. tuberculosis: Mycobacterium tuberculosis; TB: Tuberculosis; MDR: Multi-drug resistance; INH: Isoniazid; INH-R: Isoniazid resistance; XDR: Extensive drug-resistance.

## Competing interests

The authors declare that they have no competing interests.

## Authors’ contributions

HYP and WJK conceived the initial idea and the study design; JUS, HY, SWU, KJ, SW and HYP linked the data, contributed to data analysis and results interpretation; JUS and HY draft the manuscript; GYS, MPC, HK, OJK, NYL and HYP revised manuscript. All authors read and approved the final manuscript.

## Pre-publication history

The pre-publication history for this paper can be accessed here:

http://www.biomedcentral.com/1471-2334/13/404/prepub
